# Pharmacist-led olaparib follow-up service for ambulatory ovarian cancer patients: A prospective study in a tertiary specialized cancer hospital in China

**DOI:** 10.3389/fphar.2022.1037726

**Published:** 2022-12-13

**Authors:** Yanting Wang, Di Du, Jun Yang, Alan H. Lau, Yuanyuan Dai, Wangjun Qin, Ning Li, Guohui Li

**Affiliations:** ^1^ Department of Pharmacy, National Cancer Center/National Clinical Research Center for Cancer/Cancer Hospital, Chinese Academy of Medical Sciences and Peking Union Medical College, Beijing, China; ^2^ Pharmaceutical Department, Hubei Cancer Hospital, Tongji Medical College, Huazhong University of Science and Technology, Wuhan, Hubei, China; ^3^ Department of Pharmacy Practice, College of Pharmacy, University of Illinois at Chicago, Chicago, IL, United States; ^4^ National Cancer Center/National Clinical Research Center for Cancer/Hebei Cancer Hospital, Chinese Academy of Medical Sciences, Langfang, China; ^5^ Department of Pharmacy, China-Japan Friendship Hospital, Beijing, China; ^6^ Department of Gynecologic Oncology, National Cancer Center/National Clinical Research Center for Cancer/Cancer Hospital, Chinese Academy of Medical Sciences and Peking Union Medical College, Beijing, China

**Keywords:** olaparib, ovarian cancer, pharmacist, follow-up, adverse drug reactions, drug adherence, drug-drug interaction

## Abstract

**Purpose:** To establish a pharmacist-led olaparib follow-up program for ovarian cancer patients, provide patient education, get information on adverse drug reactions (ADRs), and identify and manage drug-related problems.

**Methods:** Ambulatory adult patients with ovarian cancer receiving olaparib were enrolled. At least one follow-up session was conducted by clinical pharmacists. Pharmacists collected data on the type and grade of ADRs, drug adherence, olaparib dosing, concomitant medications, and pharmacists’ suggestions.

**Results:** 83 patients were enrolled with the median age of 58. The average number of the follow-up sessions provided to each patient was 1.31, and the average duration of each follow-up was 17.78 min. The olaparib starting dose for most patients (97.59%) was 600 mg/d. 36.14% of the patients had missed olaparib doses and 27.71% of the patients had dose adjustments due to ADRs. The most common ADRs (incidence≥10%) were: fatigue (40.96%), anemia (36.14%), leukopenia (36.14%), nausea (28.92%), thrombocytopenia (16.87%), anorexia (16.87%), dyspepsia (15.66%). The tolerability profiles were generally similar between patients treated for “first-line maintenance” and those treated for “recurrence maintenance” (*p* > .05). There were 42% of the patients who were concomitantly taking medications without exact chemical contents (such as formulated Chinese medicines and Chinese decoctions), and common types of concomitant medications with exact drug names were antihypertensive, anti-hyperglycemic, and anti-hyperlipidemic medications. The pharmacists identified 4 clinically significant drug-drug interactions (DDIs) in two patients. Pharmacists made 196 suggestions mainly related to rational use of the medications and management of ADRs.

**Conclusion:** The study provides the first report about pharmacist-led follow-up service for olaparib. The types of ADRs were similar to those previously observed in clinical trials, and the profiles of ADRs in different types of patients (first-line maintenance vs. recurrence maintenance) were also similar. Pharmacists identified drug-related problems (such as adherence, DDIs and management of ADRs) and offer suggestions for the patients.

## Introduction

Ovarian cancer is a common gynecological cancer worldwide. The majority of ovarian cancers are epithelial ovarian cancer (EOC), and most patients are diagnosed as FIGO (International Federation of Gynecology and Obstetrics) III/IV (Ni et al., 2019). The traditional standard treatment for EOC is maximal cytoreductive surgery and platinum-based chemotherapy. However, about 80% of the patients experienced relapse within 1–2 years (Kim et al., 2012).

In recent years, olaparib, a poly ADP-ribose polymerase (PARP) inhibitor, provides a new modality for treating ovarian cancer. Substantial clinical benefit has been shown in trials such as SOLO1 (Moore et al., 2018), SOLO 2 (Poveda et al., 2020), and Study 19 (Friedlander et al., 2018). Olaparib is the first-class inhibitor of PARP enzymes, including PARP1, PARP2 and PARP3. Olaparib has been approved by US Food and Drug Administration (FDA) for the following indications: 1) maintenance treatment of recurrent ovarian cancer (including epithelial ovarian, fallopian tube or primary peritoneal cancer) in adults who are in complete or partial response (CR or PR) to platinum-based chemotherapy (also called “recurrence maintenance”); 2) first-line maintenance treatment for deleterious or suspected deleterious somatic or germline BRCA-mutated (sBRCAm or gBRCAm) advanced ovarian cancer patients who are in CR or PR to first-line platinum-based chemotherapy (also called “first-line maintenance”); 3) first-line maintenance treatment for advanced ovarian cancer in combination with bevacizumab for adult patients who are in CR or PR to first-line platinum-based chemotherapy and with homologous recombination deficiency (HRD) positive status; and 4) treatment of adults with deleterious or suspected deleterious gBRCAm advanced ovarian cancer who have been treated with ≥3 prior lines of chemotherapy (US Food And Drug Administration, 2020). In China, olaparib is approved only for the first two indications.

Patients treated with oral antitumor agents are likely to have less frequent contact with healthcare professionals. As a result, patient safety, drug adherence, medication therapy monitoring, and timely follow-up might be compromised (Krikorian et al., 2019). As shown in SOLO-1 trial, SOLO-2 trial and Study 19, commonly seen olaparib-induced adverse drug reactions (ADR) were fatigue (67%, 66% and 63% respectively), anemia (38%, 44% and 23%), thrombocytopenia (11%, 14% and 4%), neutropenia (17%, 19% and 5%), nausea (77%, 76% and 71%), vomiting (40%, 37% and 35%), diarrhea (37%, 33% and 28%), and dysgeusia (26% and 27% respectively for Solo-1 and Solo-2 trials, not reported in Study 19). The consequences of severe adverse reactions are usually catastrophic, in particular, hematological ADRs are typically difficult to identify, especially in the absence of regular monitoring. Most of the patients receiving olaparib are middle-aged or elderly who commonly receive different medications for chronic conditions, such as anti-hypertensives, anti-hyperglycemics and anti-hyperlipidemia medications. Therefore, patients would benefit from regular professional evaluation and guidance on potential drug-drug interactions (DDIs) as well as other drug-related problems.

Oncology clinical pharmacists, as members of the healthcare team, are playing important roles in delivering care (such as drug selection, dose adjustment, ADR monitoring, identification and management of DDIs etc.) (Riu-Viladoms et al., 2019; Wang et al., 2020a; Wang et al., 2020b). Recently, oncology pharmacists have demonstrated their contributions in optimizing treatment outcomes and enhancing patient satisfaction in follow-up programs for oral antitumor drugs (Bertsch et al., 2016; Ribed et al., 2016; Riu-Viladoms et al., 2019; Collado-Borrell et al., 2020; Khrystolubova et al., 2020). They have also assumed active roles in developing clinical guidelines and expert consensuses on safe and rational medication use in oncology practice (Holle and Boehnke, 2014; Li et al., 2019a; Li et al., 2019b; Li et al., 2019c; Yang et al., 2020).

Olaparib was approved at the end of 2018 in China. So far, little has been reported about the services provided by oncology pharmacists during olaparib treatment. In order to address this challenge, an oncology pharmacist-driven follow-up program was established to provide pharmaceutical care for ambulatory ovarian cancer patients (including epithelial ovarian, fallopian tube or primary peritoneal cancer patients) receiving olaparib in a tertiary cancer-specialized hospital in China. The aim of this study led by pharmacists was to establish a follow-up program for ovarian cancer patients receiving olaparib, provide patient education, get information on adverse drug reactions (ADRs), identify and manage drug-related problems.

## Materials and methods

Inclusion criteria: Female ambulatory patients (≥18 years old) with ovarian, fallopian tube, or primary peritoneal cancers who were receiving olaparib.

Exclusion criteria: Patients who were not able to or unwilling to answer follow-up calls.

**FIGURE 1:**
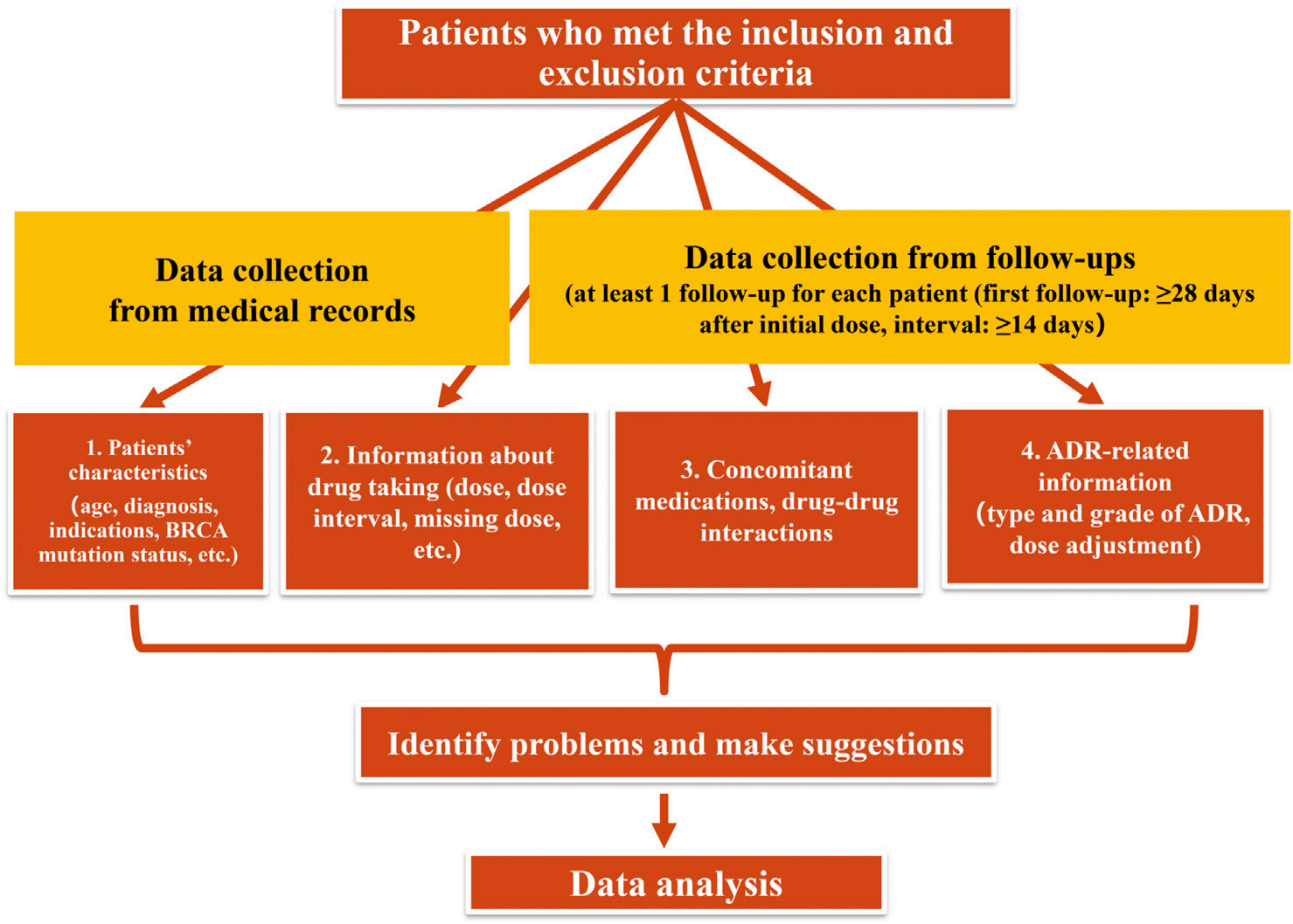
Flowchart of the study.

In this prospective study conducted from November 2019 to March 2021, the clinical pharmacists made at least one follow-up telephone call for each eligible patient during their treatment, the following data were obtained during the follow-up: 1) dosage and frequency of olaparib; 2) changes in olaparib dosing; 3) type and grade of olaparib-induced ADRs; 4) drug adherence (assessed by asking whether patients had any missing doses); 5) concomitant medications and clinically significant DDIs and drug-food interactions; 6) type and number of suggestions to the patients made by the clinical pharmacists; 7) duration of the follow-up; 8) number of follow-up calls for each patient. Additional medical information, such as age, diagnosis, BRCA mutation status, etc. were collected from electronic medical records. The flowchart of the follow-up was described in Figure 1.

Two well-trained independent pharmacists performed the causality assessment of ADRs using the World Health Organization-Uppsala Monitoring Centre causality scale (WHO-UMC causality scale). The DDIs were assessed by two independent pharmacists and the assessment was mainly based on databases “UpToDate” and “Drugs.com”.

### Statistical analysis

Data processing was performed using SPSS 22.0 statistical software (SPSS Inc., Chicago, USA). All data are expressed as numbers (n) or percentages (%). Categorical variables were analyzed with the χ test. A P value of <0.05 was defined as statistically significant.

## Results

### Patient’s characteristics

Eighty three patients were enrolled in the study and included in the analysis. The total number of the follow-up phone calls were 109, and the total duration of all the follow-up calls were 1,440 min. There were 2 patients whose data for the duration of the follow-up were not recorded. On average, each patient received 1.31 follow-up calls, and the average duration was 17.78 min. Other information such as age, diagnosis, BRCA mutation status, and the initial dose of olaparib is shown in Table 1.

**TABLE 1:** Patients’ characteristics.

Item	N (%)
Total number of patients	83 (100%)
Age (years)	
<60	48 (57.83%)
≥60	35 (42.17%)
Median age (range)	58 (35–82)
Diagnosis	
Ovarian cancer	81 (97.59%)
Fallopian tube cancer	1 (1.20%)
Ovarian and fallopian tube cancer	1 (1.20%)
BRCA status	
BRCA1 mutation	24 (28.92%)
BRCA2 mutation	7 (8.43%)
BRCA1/2 mutation (not recorded exactly)	3 (3.61%)
No mutation	15 (18.07%)
Not known	34 (40.96%)
Indication for olaparib	
Maintenance treatment after first-line chemotherapy	23 (27.71%)
Maintenance treatment after recurrence	58 (69.88%)
Unknown	2 (2.41%)
The initial dose of olaparib	
600mg/d	81 (97.59%)
450mg/d	2 (2.41%)

### Drug adherence

There were 36.15% of patients had missed one or more doses of olaparib. Most patients missed doses because they forgot to take the drug, while some patients missed doses intentionally because they had concerns about potential DDIs. For example, one patient who was receiving concurrent ibuprofen intentionally missed one olaparib dose every day because she was afraid of the potential DDI between ibuprofen and olaparib. For patients who had missed doses, we asked patients for the exact reasons for missing the doses, educated patients about the importance of adherence, and made the following suggestions: 1) use tools to aid adherence (such as pill boxes, alarms, calendars, daily routines); 2) ensure timely orders for refill; 3) confirm with physicians or pharmacists when they have to take additional medications and have concerns about DDIs.

### Tolerability profiles of olaparib-induced adverse drug reactions

In this study, the most common ADRs (incidence≥10%) were: fatigue (40.96%), anemia (36.14%), leukopenia (36.14%), nausea (28.92%), thrombocytopenia (16.87%), anorexia (16.87%), dyspepsia (15.66%). The incidence of all the ADRs (all grades) and all the severe ADRs (grades 3–4) were tabulated in Table 2. There were 27.71% of the patients who had dose adjustments due to ADRs. In addition, we also compared the ADR profiles among different groups of patients, and the result showed that the tolerability in patients treated with olaparib for “first-line maintenance” was not different with those for “recurrence maintenance” (Table 3).

**TABLE 2:** Incidence of olaparib-induced ADRs.

Adverse drug reactions	All grades	Incidence (%)	Grades 3/4	Incidence (%)
Fatigue	34	40.96	3	3.61
Anemia	30	36.14	6	7.23
Leukopenia	30	36.14	2	2.41
Nausea	24	28.92	0	0.00
Thrombocytopenia	14	16.87	2	2.41
Anorexia	14	16.87	0	0.00
Dyspepsia	13	15.66	0	0.00
Pain (excluding gastrointestinal pain and headache)	8	9.64	0	0.00
Oral mucositis	7	8.43	1	1.20
Vomiting	6	7.23	0	0.00
Dysgeusia	5	6.02	0	0.00
Rash maculo-papular	4	4.82	0	0.00
Constipation	3	3.61	0	0.00
Dizziness	3	3.61	0	0.00
Insomnia	3	3.61	0	0.00
Gastrointestinal pain	2	2.41	0	0.00
Headache	2	2.41	0	0.00
Hypersomnia	2	2.41	0	0.00
Hypotension	2	2.41	0	0.00
Aminotransferase increased	2	2.41	0	0.00
Diarrhea	1	1.20	0	0.00
Edema limbs	1	1.20	0	0.00
Alopecia	1	1.20	0	0.00
Creatinine increased	1	1.20	0	0.00
Tachycardia	1	1.20	0	0.00

Adverse drug reactions were graded according to National Cancer Institute Common Terminology Criteria for Adverse Events (NCI CTCAE), version 5.0.

**TABLE 3:** The tolerability profile among patients receiving olaparib due to different lines of treatment.

	Number (%)	Indications of olaparib			F	*P* Value
		Maintenance after first-line chemotherapy	Maintenance after recurrence chemotherapy	Not known		
Blood and lymphatic system disorders	—	—	—	—	1.373	0.547
With	47 (56.63%)	12	33	2	—	—
Without	36 (43.37%)	11	25	0	—	—
Gastrointestinal disorders	—	—	—	—	3.011	0.177
With	43 (51.80%)	10	33	0	—	—
Without	40 (48.20%)	13	25	2	—	—
Metabolism and nutrition disorders	—	—	—	—	1.706	0.537
With	14 (16.87%)	2	12	0	—	—
Without	69 (83.17%)	21	46	2	—	—
General disorders	—	—	—	—	2.491	0.235
With	39 (46.99%)	12	25	2	—	—
Without	44 (53.01%)	11	33	0	—	—
Nervous system disorders	—	—	—	—	2.617	0.299
With	11 (13.25%)	3	7	1	—	—
Without	72 (86.75%)	20	51	1	—	—
Skin and subcutaneous tissue disorders	—	—	—	—	0.778	1.000
With	5 (6.02%)	1	4	0	—	—
Without	78 (93.98%)	22	54	2	—	—
Miscellaneous	—	—	—	—	5.500	0.066
With	8 (9.64%)	1	5	2	—	—
Without	75 (90.36%)	22	53	0	—	—

The classification of adverse drug reactions was based on National Cancer Institute Common Terminology Criteria for Adverse Events (NCI CTCAE), version 5.0.

### Concomitant medications and DDIs

The pharmacists recorded all the concomitant medications (including patent and generic medications, formulated Chinese medicine and Chinese decoctions, and food supplements) and evaluated potential DDIs for all the patients in the study. 57 patients were found to have 81 types of concomitant medications (Table 4). The pharmacists identified four clinically significant DDIs, involving 4 types of medications in two patients, and provided suggestions for management (Table 5). It is important to note that some patients were taking concomitant Chinese decoction or formulated Chinese medicine, whose interactions with olaparib were unknown or difficult to evaluate (due to the complexity of their contents). The pharmacists therefore recommended patients to take olaparib and Chinese decoction (or formulated Chinese medicine) separately, with at least 2 h in-between to try to avoid potential DDIs.

**TABLE 4:** Concomitant medications in patients receiving olaparib.

Concomitant medications	Types of medications (%)
Anti-tumor medications	5 (6.2%)
Altretamine	1
Letrozole	1
Thalidomide	1
Megestrol	1
Apatinib	1
Medications for chronic diseases	42 (51.9%)
Anti-hypertensive medications	17
Anti-hyperglycemic medications	6
Anti-hyperlipidemic medications	3
Anti-platelet medications	1
Neuropsychiatric medications	3
Others	12
Other medications with unknown potential for DDIs or without specific name (or without exact chemical contents)	34 (42.0%)
Health products (or food supplements)	2
Medications without exact name	6
Formulated Chinese medicines	16
Chinese decoction	10

**TABLE 5:** DDIs identified by the pharmacists.

Concomitant medications	Risk rating	Reason for the interaction	Suggestions made by the pharmacists
Acarbose and hydrochlorothiazide	C (monitor therapy)	Hydrochlorothiazide may diminish the therapeutic effect of acarbose	Closely monitor blood glucose by lab test
Metformin and hydrochlorothiazide	C (monitor therapy)	Hydrochlorothiazide may diminish the therapeutic effect of metformin	Closely monitor blood glucose by lab test
Glimepiride and acarbose	D (consider therapy modification)	Acarbose may enhance the hypoglycemic effect of glimepiride	Closely monitor blood glucose by lab test and to have an endocrinologist to assess the potential use of an alternative medication
Glimepiride and metformin	C (monitor therapy)	Metformin may enhance the hypoglycemic effect of glimepiride	Closely monitor blood glucose by lab test

### Suggestions made by the pharmacists

During the follow-up, the pharmacists identified drug-related problems and made 196 suggestions to the patients. Some patients were not aware of the importance and the frequency of obtaining lab tests to monitor ADRs, and we suggested them that the frequency could be once every week at the beginning of olaparib treatment, and then once every 2–4 weeks when stable. Some patients had developed grade 2 or higher ADRs, and we suggested them to contact physicians for dose adjustments. Some patients were taking olaparib with a wrong frequency, and we suggested them to take it twice a day with the best frequency of 12 h. Some patients were not aware that limes and grapefruits should not be taken concomitantly with olaparib, and we suggested that it was best not to eat these fruits during the treatment and explained the underlying reasons. The nature/types of the suggestions as well as the number and percentage for each type are tabulated in Table 6.

**TABLE 6:** Suggestions made by the pharmacists.

Item	Number of suggestions (%)
Recommendations on obtaining relevant lab test to monitor ADRs on a regular basis	51 (26.02%)
Recommendations on rational use and contacting physicians for dosage adjustment for olaparib	36 (18.37%)
Recommendations on seeking medical care	25 (12.76%)
Recommendations on drug-food interactions	23 (11.73%)
Recommendations on modification of eating habits for preventing specific ADRs	23 (11.73%)
Recommendations on DDIs	18 (9.18%)
Recommendations on management of ADRs	13 (6.63%)
Recommendations on life style modification for preventing specific ADRs	7 (3.57%)
Total number of suggestions	**196**

### Questions raised by patients during the follow-up phone calls

During the follow-up phone calls, aside from responding to questions raised by the pharmacists, some of the patients (36/83, 43.37%) positively raised some questions about the drug. For example, some patients were curious about the duration of the treatment, and we told them that this should be determined by physicians with both evaluations of the efficacy and the ADRs. Some patients were complaining that it was difficult to refill the prescriptions during the COVID-19 pandemic and asking for our advice, and we patiently told them the ways to make appointments online or by telephone. Some patients were curious about the nature and the purpose of the follow-up, and we explained that this work was conducted to identify drug-related problems and offer professional suggestions for the patients. A total of 54 questions were raised, which have been classified into 8 types. The pharmacists answered the questions and resolved the patients’ confusion. The number and percentage of each type of the questions are tabulated in Table 7.

**TABLE 7:** Questions from patients during the follow-up sessions.

Types of the questions	Number of questions (%)
Drug information (such as ADRs, dosage, half-life, etc.)	12 (22.22%)
DDIs	3 (5.56%)
Drug-food interactions	9 (16.67%)
The nature of the follow-up performed by pharmacists	9 (16.67%)
Seeking medical care and filling prescriptions	6 (11.11%)
Treatment efficacy	4 (7.41%)
Course of the treatment	6 (11.11%)
Treatment costs (including medical insurance coverage)	5 (9.26%)
Total number of questions	**54**

## Discussion

The study provides the first report about pharmacist-led follow-up service for olaparib and portrayed several characteristics for these patients in the real world: 1) the types of ADRs were similar to those previously observed in clinical trials, and the profiles of ADRs in different types of patients (first-line maintenance vs. recurrence maintenance) were also similar; 2) there were 36.14% of the patients had missed olaparib doses, indicating drug adherence is not very good; 3) there were 42% of the patients who were concomitantly taking medications without exact chemical contents (such as formulated Chinese medicines and Chinese decoctions), and common types of concomitant medications with exact drug names were antihypertensive, anti-hyperglycemic, and anti-hyperlipidemic medications; 4) the pharmacists identified 4 clinically significant Drug-drug interactions (DDIs) in two patients; 5) pharmacists made 196 suggestions mainly related to rational use of the medications and management of ADRs.

In this study, we found that the most common ADRs (incidence≥10%) were: fatigue, anemia, leukopenia, nausea, thrombocytopenia, anorexia, dyspepsia. Most of the patients experienced grades 1–2 ADRs. 16.87% (14/83) of the patients developed grades 3–4 ADRs, including severe fatigue, anemia, leukopenia, thrombocytopenia and oral mucositis. In general, these ADRs were manageable, as indicated by the relatively low proportion of patients who required olaparib dosage adjustment due to the ADRs (23/83, 27.71%). Previously, in a pooled safety analysis with data from 2,351 patients (1,585 patients among them were exposed to 300 mg olaparib tablet, twice a day, and 766 patients were exposed to 400 mg olaparib capsule, twice a day), the types of the most common adverse reactions in ≥10% of the patients were similar to those reported in our study (US Food And Drug Administration, 2020). Additionally, we have been the first to report that there was no significant difference of the incidence of ADRs between patients who used olaparib as “first-line maintenance” and those used it as “recurrence maintenance”, indicating that the methods of ADR management in these groups of patients could be generally the same.

Adherence is defined as the degree to which one conforms to provider’s instructions on day-to-day treatment with respect to the timing, dosage, and frequency (Cramer et al., 2008). Adherence is important in improving outcomes of chronic diseases and has been shown to be associated with reduction in healthcare costs (Sokol et al., 2005). There are several ways (direct, indirect, indirect and subjective ways) to measure adherence, but none of them has been considered a “gold standard”. The direct ways are either direct observation or measurement of serum drug levels. The indirect ways include 1) pill counts; 2) microelectronic event monitoring system; 3) refill records; 4) biomarkers; and 5) outcomes. The indirect and subjective ways are 1) self-report; 2) others’ assessment; and 3) diaries (Geynisman and Wickersham, 2013). In our study, we used “self-report” as the single indicator for adherence. Our results show that 36.15% of patients had missed one or more doses of olaparib, indicating that patient education about the importance of adherence is very necessary. Besides, it is worth noting that it could be more objective to evaluate adherence with combined ways in further studies.

Olaparib is primarily metabolized by CYP 3A4/5. Potent CYP3A inhibitors (such as itraconazole, clarithromycin, voriconazole, lopinavir/ritonavir) and potent CYP3A inducers (such as phenytoin, rifampicin, carbamazepine) should not be used concomitantly with olaparib. Additionally, food containing CYP3A inhibitors should also be avoided, such as grapefruit (or grapefruit juice) and lime (or lime juice). In this study, there was no patient concomitantly using potent CYP3A inhibitors or inducers, and we only identified four clinically significant DDIs. However, it is worth noting that many patients were taking concomitant Chinese decoction or formulated Chinese medicine, whose interactions with olaparib were unknown or difficult to evaluate (due to the complexity of their contents). This is a very common and important phenomena in Chinese patients. Further study could be focused on looking for ways to get to know the full formula of the Chinese decoctions and develop databases for analyzing DDIs among Chinese decoctions and western medicines.

In addition to evaluating potential DDIs and making related recommendations, the pharmacists also initiated other suggestions that were important to optimize treatment, such as: 1) reminding patients the need to regularly monitor ADRs with relevant lab tests at a clinic; 2) making recommendations to patients on rational drug usage and contacting physicians for dosage adjustment; 3) giving advice on avoiding potential drug-food interactions; 4) making recommendations to patients on management of ADRs. It is worth noting that all the recommendations were provided directly to the patients. The reasons for this are that, 1) in China, pharmacists have no prescription rights; 2) the number of outpatients is very big in large hospitals; 3) outpatients in the same department are from many different physicians. For these reasons, when pharmacists identify that there is a need for patients, especially outpatients, to change the prescription, they usually suggest them to directly contact the physicians, rather than making suggestions to the physicians. The shortcoming of this is that it is not convenient to evaluate the roles of pharmacists (e.g., the acceptance rate of the suggestions made by the pharmacists to physicians). However, in some countries, pharmacists work with physicians in many kinds of collaborative programs under specific agreements, in this case the impact of services provided by pharmacists could be better demonstrated. For example, Conliffe and colleagues reported a pharmacist-run oral antineoplastic monitoring program and its impact on improving therapy adherence. In this program, pharmacists made clinically significant interventions and received high patient satisfaction, providing justification for service expansion into other disease states (Conliffe et al., 2019). Khrystolubova and colleagues reported a pharmacist-led, multi-center, collaborative patient education and proactive ADR management program in a community-based oncology setting. They showed that ADRs in patients with EGFRm+ non-small cell lung cancer receiving afatinib were well managed by this pharmacist-led service (Khrystolubova et al., 2020). Suzuki and colleagues collaborated with medical oncologists to establish an integrated support program aimed at preventing unnecessary treatment interruption or dose reduction during oral lenvatinib targeted therapy. They showed that the interventions provided by pharmacists and medical oncologist improved lenvatinib therapy, by adding supportive medications for management of ADRs and correcting mistakes in taking medications (Suzuki et al., 2020). Hansen and colleagues reported a Collaborative Drug Therapy Management (CDTM) program in the gynecologic oncology clinic, and they demonstrated that pharmacists (with authorities to order lab tests and prescribe certain medications in accordance with CDTM program agreements) managed chemotherapy-related adverse reactions and provided therapeutic interventions. Additionally, both patients and physicians reported that such collaborative services were valuable (Hansen et al., 2016). To improve our service, we are now planning to establish a physician-pharmacist collaborative program for ambulatory patients, in which pharmacists and physicians would work closer to provide services to patients in a real-time fashion, and share professional opinions with each other in a more convenient way.

## Limitations

First, the sample size of the study is relatively small. Second, only one indicator was used to evaluate patients’ adherence. Third, the direct impact and the roles of pharmacists could be better demonstrated by having a comparison group of patients without follow-up service conducted by pharmacists.

## Conclusion

The study provides the first report about pharmacist-led follow-up for olaparib. The types of ADRs were similar to those previously observed in clinical trials, and the profiles of ADRs in different types of patients (first-line maintenance vs. recurrence maintenance) were also similar. Pharmacists identified drug-related problems (such as adherence, DDIs and management of ADRs) and offer suggestions for the patients.

## Data Availability

The original contributions presented in the study are included in the article/Supplementary Material, further inquiries can be directed to the corresponding authors.
